# Global and regional prevalence of disabilities among children and adolescents: Analysis of findings from global health databases

**DOI:** 10.3389/fpubh.2022.977453

**Published:** 2022-09-23

**Authors:** Bolajoko O. Olusanya, Vijaya Kancherla, Amira Shaheen, Felix A. Ogbo, Adrian C. Davis

**Affiliations:** ^1^Centre for Healthy Start Initiative, Lagos, Nigeria; ^2^Department of Epidemiology, Emory University Rollins School of Public Health, Atlanta, GA, United States; ^3^Division of Public Health, Faculty of Medicine and Health Sciences, An-Najah National University, Nablus, Palestine; ^4^Translational Health Research Institute (THRI), Western Sydney University, Penrith, NSW, Australia; ^5^Department of Population Health Science, London School of Economics, London, United Kingdom; ^6^Vision and Eye Research Institute, School of Medicine Anglia Ruskin University, Cambridge, United Kingdom

**Keywords:** developmental disabilities, functional impairments, global health, Global Burden of Disease, statistical modeling, low-income and middle-income countries, SDGs, ICF

## Abstract

**Objective:**

The United Nations' Sustainable Development Goals (SDGs) require population-based data on children with disabilities to inform global policies and intervention programs. We set out to compare the prevalence estimates of disabilities among children and adolescents younger than 20 years as reported by the world's leading organizations for global health statistics.

**Methods:**

We purposively searched the disability reports and databases of the United Nations Children's Fund (UNICEF), the World Health Organization (WHO), the World Bank and the Global Burden of Diseases (GBD) Study. We analyzed the latest disability data reported by these organizations since 2015. We examined the methodologies adopted in generating the reported prevalence estimates and evaluated the degree of agreement among the data sources using Welch's test of statistical difference, and the two one-sided *t-*test (TOST) for statistical equivalence.

**Results:**

Only UNICEF and GBD provided the most comprehensive prevalence estimates of disabilities in children and adolescents. Globally, UNICEF estimated that 28.9 million (4.3%) children aged 0–4 years, 207.4 million (12.5%) children aged 5–17 years and 236.4 million (10.1%) children aged 0–17 years have moderate-to-severe disabilities based on household surveys of child functional status. Using the UNICEF estimated prevalence of 10.1%, approximately 266 million children aged 0–19 years are expected to have moderate-to-severe disabilities. In contrast, GBD 2019 estimated that 49.8 million (7.5%) children aged under 5 years, 241.5 million (12.6%) children aged 5–19 years and 291.3 million (11.3%) children younger than 20 years have mild-to-severe disabilities. In both databases, Sub-Saharan Africa and South Asia accounted for more than half of children with disabilities. A comparison of the UNICEF and GBD estimates showed that the overall mean prevalence estimates for children under 5 years were statistically different and not statistically equivalent based on ±3 percentage-point margin. However, the prevalence estimates for children 5–19 years and < 20 years were not statistically different and were statistically equivalent.

**Conclusion:**

Prevalence estimates of disabilities among children and adolescents generated using either functional approach or statistical modeling appear to be comparable and complementary. Improved alignment of the age-groups, thresholds of disability and the estimation process across databases, particularly among children under 5 years should be considered. Children and adolescents with disabilities will be well-served by a variety of complementary data sources to optimize their health and well-being as envisioned in the SDGs.

## Introduction

The disability-inclusive provisions in the Sustainable Development Goals (SDGs) require policy interventions to address the needs of children with disabilities and bridge the inequalities that exist between children with and without disabilities (1). However, unlike child mortality which has improved substantially since 2000 (2), reliable global estimates of children with disabilities have been lacking, a situation that has often been misconstrued as evidence that disability is not an important or a serious enough public or global health issue (3). For many years, the absence of consensus on the definition and measurement of disability to facilitate comparable data cross-nationally has been a major challenge in generating the necessary estimates (4). Estimates generated traditionally from systematic reviews and meta-analyses of specific disabilities are often unusable to justify global initiatives because of substantial variations in the quality and methodologies of the underlying studies including the poor representation of high-burden populations from low- and middle-income countries (5, 6). These reservations have accounted for the growing reliance by policymakers on alternative approaches and sources of global estimation of population health metrics including household surveys and statistical modeling (6).

In 2001, the World Health Organization (WHO) launched the International Classification of Functioning, Disability and Health (ICF) to standardize the evaluation of disabilities over the life course (7). In the same year, the Washington Group on Disability Statistics (Washington Group) was commissioned under the auspices of the United Nations (UN) to develop suitable disability measures that will facilitate comparable disability data within the ICF framework (8). In 2006, the Convention on the Rights of Persons with Disabilities (CRPD) provided an operational definition for children with disabilities as “children 18 years or younger who have ‘long-term physical, mental, intellectual, or sensory impairments which in interaction with various barriers may hinder their full and effective participation in society on an equal basis with others.” (9). In the same year, some 150 million children were estimated by the United Nations Children's Fund (UNICEF) to have disabilities (10). However, no details were provided on how this estimate was generated, and the age range of children included.

In the first World Health Report on Disability published by WHO in 2011, 93 million children (0–15 years) were estimated to have a moderate-to-severe disability, and 13 million had a severe disability (11). These estimates were based on statistical modeling of limited data sources by the Global Burden of Diseases, Injuries, and Risk Factors Study (GBD) 2004, and they excluded children with mild but functionally disabling impairments which was inconsistent with the ICF framework (7). Additionally, the proportion of children under 5 years with disabilities, who were likely to benefit most from early childhood intervention services, was not reported. While these WHO estimates were reported with reservation by UNICEF (12), they were widely cited in the literature and by several UN agencies until an update was published in 2020 based on the GBD 2017 data (13).

Several provisions of the SDGs for disability issues, especially for inclusive education (SDG 4), now make it imperative to generate estimates of children with disabilities (1). These provisions are reinforced by the urgent need to address the disturbing disparities between the global trends in mortality and morbidity among children and adolescents since 2000 (14, 15). This article, therefore, set out to analyze the global and regional estimates of children and adolescents younger than 20 years with disabilities published in global health databases since the launch of the SDGs in 2015.

## Methods

### Data sources and approaches to disability estimation

For this study, we purposively searched disability reports and databases of UNICEF, WHO, the World Bank and the GBD produced by the Institute for Health Metrics and Evaluation (IHME), as these are presently the leading sources of population-based data for research and policy decisions in global health. The methodological approaches used by these databases were examined to provide context for the reported prevalence estimates of disabilities in children and adolescents. For the remainder of this study, the term “children with disabilities” refers to “children and adolescents with disabilities” below the age of 20 years, except otherwise stated. We relied entirely on the data available to the public and did not contact the organizations for any additional information. A summary of the key features of the available data sources is presented in Table 1.

**Table 1:** Summary of data sources for global estimates of disabilities in children and adolescents.

	**UNICEF**	**WHO-World Bank**	**IHME**	**WHO-IHME**
Title	Multiple Indicator Cluster Survey (MICS)	Model Disability Survey (MDS)	Global Burden of Disease (GBD)	WHO Rehabilitation Need Estimator
Disability model	Biopsychosocial/ICF	Biopsychosocial/ICF	Medical	Medical
Disability measurement	Parent (or household member)-reported functional difficulties	Parent (or household member)-reported functional difficulties and known impairments	Diagnosis of impairments based on the International Classification of Diseases (ICD) codes	Diagnosis of impairments based on the International Classification of Diseases (ICD) codes
Sources of data input	Household surveys	Household surveys	Systematic reviews of the literature, hospital and claims databases, health surveys, case notification systems, cohort studies, and multinational survey data	Systematic reviews of the literature, hospital and claims databases, health surveys, case notification systems, cohort studies, and multinational survey data Rehabilitation experts
Measurement tool(s)	UNICEF/Washington Group Child Functioning Module, Washington Group Short Set on Functioning, and Global Activity Limitation Indicator	Children version of the Model Disability Survey (MDS) questionnaire	Statistical modelling of sequelae of health conditions	Statistical modelling of sequelae of health conditions
Age group(s)	• 2–4 years • 5–17 years	• < 5 years • 5–12 years • 13–17 years	0–19 years	0–19 years
Countries covered	43	Not available	193	193
Included in analysis	Yes	No, data collection on-going	Yes	No. fewer impairments in children reported

UNICEF, United Nations Children's Fund; IHME, Institute for Health Metrics and Evaluation; WHO: World Health Organization.

ICF, International Classification of Functioning, Disability and Health.

#### UNICEF Disability Report 2022

In 2016, UNICEF in partnership with the Washington Group developed a Child Functioning Module (CFM) for inclusion in its routine Multiple Indicator Cluster Survey (MICS) implemented worldwide (16, 17). The CFM appears to conform largely with the biopsychosocial model of disability, by focusing on the presence and extent of functional difficulties rather than on body structure or conditions. It consists of two questionnaires, one with 16 questions for children aged 2–4 years and the other with 24 questions for children aged 5–7 years. The questionnaires are designed to assess functional difficulties in 8 developmental domains of hearing, vision, mobility, fine motor, communication/comprehension, emotions, learning, and playing; and are administered to mothers and primary care givers of eligible children. Responses reflect different levels of severity measured on a 4-level Likert rating scale (0 = no difficulty, 1 = some difficulty, 2 = a lot of difficulty and 3 = cannot do at all). This scale allows the proportion of children with mild difficulties (those who respond “at least some difficulty”), or moderate difficulties (those who respond “a lot of difficulty”) or those with severe difficulties (those who respond “cannot do at all”) on one or more domain of functioning to be estimated. For reporting purposes, a child with a disability is considered as one with a score level of 3 or 4 in one or more of the 8 functional domains, which meant that children with the mildest degrees of difficulty are excluded. In the UNICEF report first published in 2021, data were collected from 103 data sources (across 43 countries and areas) representing 84 per cent of the world's population of children and at least 50 per cent of population of children in each world region (https://data.unicef.org/resources/children-with-disabilities-report-2021/). Data were first-of-all collected using three different instruments: UNICEF/Washington Group Child Functioning Module, Washington Group Short Set on Functioning and Global Activity Limitation Indicator; and later harmonized (17). After data harmonization, and due to significant variability across countries and regions, a meta-analytical technique was used to estimate the prevalence rates of children with disabilities for each country, 95% confidence intervals (CI) and the child population for all age groups. The estimates for children under 2 years were extrapolated from the estimates computed for children aged 2 to 4 years. It is important to clarify that the results do not provide epidemiological characteristics of any disease or impairment; rather, they provide an indication of the prevalence of moderate-to-severe functional difficulties that, in interaction with various barriers, can place children at increased risk for non-participation and exclusion.

#### The World Bank and WHO

The WHO and the World Bank Group developed the Model Disability Survey (MDS) tool in 2011 for collecting data on functioning and disability based on ICF framework (18). It is primarily designed as a standalone household survey for adults, with a shorter version to be integrated in health and other population surveys to readily facilitate the continuous monitoring of functioning and disability in a region or a country. There is an optional module for children which uniquely makes additional provision for eliciting information on health conditions, diagnosis, and treatment from the respondents. However, no global or regional estimates of children with disabilities have been published yet from the MDS. In 2020, WHO collaborated with IHME to produce the first-ever estimates of persons who experience a health condition over the course of their life that would benefit from rehabilitation based on the substantive GBD 2019 database (19, 20). The customized database was titled WHO Rehabilitation Need Estimator. A group of experts in the field of rehabilitation was convened by WHO to select specific health conditions in all age groups for which rehabilitation is a key intervention as part of an overall management plan. A total of 25 health conditions were selected for inclusion into this database. The selection for the first time included cerebral palsy as a distinct entity in the GBD database but excluded epilepsy and attention-deficit/hyperactivity disorder which were included in prior reports of children with developmental disabilities and the substantive GBD 2019 database (21, 22). For consistency, we opted to consider the six developmental disabilities reported in the substantive GBD 2019 database (https://vizhub.healthdata.org/gbd-results/). Moreover, this decision allowed the inclusion of all children with developmental disabilities regardless of expert opinion on the need for rehabilitation. The disabilities included are hearing loss, vision loss, developmental intellectual disability, epilepsy, autism spectrum disorders and attention-deficit/hyperactivity disorder. As previously reported, GBD estimates of children with developmental intellectual disability include a high proportion of children with cerebral palsy (21). The rehabilitation needs of children younger than 5 years with cerebral palsy and intellectual disability have also been reported previously (23).

#### GBD 2019 by IHME

The details of the methodologies for the six developmental disabilities selected have been extensively reported (13, 21, 22). In summary, the case definitions and diagnostic criteria were based on the WHO's global standard for diagnostic health information - International Classification of Diseases (ICD) codes (ICD-9 and ICD-10) - complemented with relevant guidelines, such as the Diagnostic and Statistical Manual of Mental Disorders (DSM)-IV-TR and the Guidelines for Epidemiologic Studies on Epilepsy (22). Hearing loss was defined as the quietest sound an individual can hear in their better ear, based on the pure-tone average (PTA) of audiometric thresholds of 0·5, 1, 2 and 4 kHz. Severity levels were classified from mild (PTA from 20 dB) to complete hearing loss (PTA > 95 dB). Vision loss was defined as an impairment resulting from all causes of moderate and worse distance vision loss, visual acuity of < 6/18 according to the Snellen chart, and uncorrected presbyopia, or near vision worse than N6 or N8 at 40 cm when best-corrected distance visual acuity was better than 6/12.

Developmental intellectual disability (or “intellectual disability” hereinafter) was defined as a condition of below-average intelligence or mental ability, with multiple severity levels. Severities were defined according to intelligence quotient (IQ) scores, ranging from borderline intellectual disability (IQ 70–85) to profound intellectual disability (IQ 0–19). Epilepsy was defined as an impairment due to idiopathic epilepsy and epilepsy secondary to known infectious and neonatal causes. This definition included cases of active epilepsy with at least one seizure in the previous 5 years, regardless of treatment. Autism spectrum disorders referred to a group of neurodevelopmental disorders with early childhood onset, incorporating disability from pervasive impairment in several areas of development, including social interaction and communication skills, plus restricted and repetitive patterns of behaviors or interests. Attention-deficit/hyperactivity disorder was defined as an externalizing disorder, incorporating disability from persistent inattention and/or hyperactivity/impulsivity using the DSM-IVTR (314.0, 314.01) and ICD-10 (F90) criteria.

In summary, the prevalence estimation for each condition started with the compilation of all available data inputs from systematic reviews of the literature, hospital and claims databases, health surveys, case notification systems, cohort studies, and multinational survey data. A comprehensive list of the sources of input data for each condition is publicly available at the Global Health Data Exchange (https://ghdx.healthdata.org/gbd-2019/data-input-sources). In the data preparation, efforts were made to i) optimize the comparability of data derived from various sources using different methods; ii) find a consistent set of estimates across prevalence data; and iii) generate estimates for locations with sparse or no data by using available information from other locations combined with covariates.

Prevalence estimates were generated using DisMod-MR 2.1, a statistical modeling technique developed specifically for the GBD project (13, 22). This is a Bayesian meta-regression tool that synthesizes epidemiological data for fatal and non-fatal health outcomes from disparate settings and sources, adjusting for different case definitions/diagnostic criteria or sampling methods, to generate internally consistent estimates by geographical location, year, age group, and sex. An overview of the analytical framework is provided in the Supplementary Figure S1 (13). Sophisticated and validated statistical modeling techniques were used to address sparse and often inconsistent data, especially for diseases, injuries, risk factors and countries for which data were insufficient (22). At every step in the modeling process, the distributions were assessed for sampling error of data inputs, the uncertainty of data corrections for measurement errors, the uncertainty in coefficients from model fit, and the uncertainty of severity distributions. Corresponding uncertainty bounds intervals (UI) for prevalence estimates were defined at the 25th and 975th value of 1,000 draws. The entire GBD process adhered to the Guidelines for Accurate and Transparent Health Estimates Reporting (GATHER), which include recommendations on documentation of data sources, estimation methods, statistical analysis, and statistical code (24).

### Statistical analysis

For our analysis, the most recent global and regional prevalence estimates of disabilities were extracted using the World Bank classification: Europe and Central Asia (ECA), East Asia and the Pacific (EAP), Eastern and Southern Africa (ESA), Latin America and the Caribbean (LAC), Middle East and North Africa (MENA), North America (NA), South Asia (SA), and West and Central Africa (WCA). A complete list of countries and areas in the regions and subregions is available at: https://data.unicef.org/regionalclassifications/. We assumed that the age groups of 0–4 years and 5–17 years used by UNICEF are comparable to the GBD age groups of under 5 years and 5–19 years, respectively. The population of children in each group that was used by UNICEF and GBD to estimate the total number of children with disabilities was compared to the official population data provided by the United Nations Population Division for each age group (25). We assessed the degree of agreement between prevalence estimates based on four criteria: statistical difference, statistical equivalence, absolute prevalence difference and prevalence ratio (26–28). Statistical difference was assessed using the Welch's *t*-test to determine the probability that the estimates from both sources are different. Statistical equivalence, which determines whether two estimates are equivalent, was explored using the two one-sided *t*-test of equivalence (TOST) based on a priori ±3 percentage-point margin typically used for comparing prevalence estimates around 10% (27). We sought to determine if the estimates for each age group were (i) statistically different and statistically equivalent, (ii) statistically different and not statistically equivalent, (iii) not statistically different and statistically equivalent, or (iv) not statistically different and not statistically equivalent (28). The absolute and relative differences were also assessed to determine whether the differences were meaningful based on *a priori* goodness-of-fit criteria of 15%, (or 0.85 to 1.15) for prevalence ratio and ≤ 5 percentage point for the absolute difference (26, 27). All tests of statistical significance were based on critical level of *p* < 0.05. The JAMOVI program for Windows version 2.2.5.0 with TOSTER module were used for analyses, as well as IBM SPSS Statistics for Windows Version 22 where possible for verification.

## Results

The disability prevalence estimates reported by UNICEF are presented in Table 2. A total of 28.9 million or 4.3% (95% CI: 4.1–4.6) of children aged 0–4 years, 207.4 million or 12.5% (95% CI: 11.7–13.3) of children aged 5–17 years, and 236.4 million or 10.1% (95% CI: 9.6–10.6) of all children aged 0–17 years were estimated to have moderate-to-severe disabilities globally. Sub-Saharan Africa (29.6% or 69.9 million) and South Asia (27.3% or 64.4 million) accounted for more than half of these children. West and Central Africa accounted for 58.7% (28.9 million) of children with disabilities in Sub-Saharan Africa. Middle East and North Africa recorded the highest prevalence (13.1%) while Europe and Central Asia had the least prevalence (5.5%) of children with disabilities. Children aged 0–4 years accounted for 12.2% of all children with disabilities.

**Table 2:** Global and regional prevalence estimates of disabilities among children younger than 18 years from UNICEF 2022.

	**Children under aged 0 to 4 years**			**Children aged 5 to 17 years**			**Children aged 0 to 17 years**		
**Region**	**%**	**95% CI**	**Number of children ('000)**	**%**	**95% CI**	**Number of children ('000)**	**%**	**95% CI**	**Number of children ('000)**
North America	4.4	3.9–4.9	943	12.0	11.3–12.7	7,073	9.9	9.5–10.4	8,016
Europe and Central Asia	2.7	2.4–3.1	1,515	6.5	5.6–7.4	9,299	5.5	4.9–6.0	10,814
East Asia and the Pacific	3.5	3.3–3.8	5,333	9.5	7.5–11.6	37,788	7.8	6.7–9.1	43,121
Latin America and the Caribbean	3.8	3.3–4.5	1,978	12.6	11.5–13.7	17,102	10.2	9.6–10.8	19,080
South Asia	3.7	2.9–4.7	6,254	13.0	10.2–16.1	58,177	10.5	9.0–12.2	64,431
Middle East and North Africa	4.5	3.3–6.0	2,246	16.9	13.5–20.5	18,694	13.1	11.3–15.1	20,940
Sub-Saharan Africa	6.0	5.2–7.0	10,648	15.9	13.3−18.6	59,300	12.7	11.2–14.3	69,948
Eastern and Southern Africa	5.2	4.5–6.0	4,509	12.8	11.2–14.4	24,356	10.4	9.5–11.3	28,865
West and Central Africa	6.8	5.8–7.9	6,139	18.9	15.3–22.7	34,944	14.9	12.8–17.2	41,083
Global	4.3	4.1–4.6	28,917	12.5	11.7–13.3	207,433	10.1	9.6–10.6	236,350

UNICEF 2022 (Reference 17).

In contrast, the GBD estimated that at least 49.8 million (7.5%) of children under 5 years (Table 3), 241.5 million (12.6%) of children aged 5–19 years (Table 4), and 291.3 million (11.3%) of all children younger than 20 years (Table 5) have mild-to-severe disabilities globally. South Asia (33.8% or 98.5 million) and Sub-Saharan Africa (20.5% or 59.8 million) accounted for more than half of these children. West and Central Africa accounted for 53.2 % (31.7 million) of children with disabilities in Sub-Saharan Africa. The highest prevalence of children with disabilities (13.6%) occurred in South Asia and the least prevalence (8.9%) in Europe and Central Asia. Children under 5 years accounted for 17.1% of all children with disabilities. Among children under 5 years, developmental intellectual disability was most prevalent (3.2%) while attention-deficit/hyperactivity disorder was least prevalent (0.2%). In contrast, among all children, hearing loss was most prevalent (4.0%), while autism spectrum disorders were the least prevalent disabilities (0.4%).

**Table 3:** Global and regional prevalence estimates (95% uncertainty intervals) of disabilities among children younger than 5 years from GBD 2019.

**Region**	**Metric**	**Hearing loss**	**Vision loss**	**Epilepsy**	**Developmental intellectual disability**	**Autism spectrum disorders**	**Attention-deficit/hyperactivity disorders**	Total^*^
North America	Number	216680 (178560–253606)	144266 (111242–184772)	116174 (86582–147558)	365935 (288933–444460)	163530 (137322–191935)	88839 (57190–129131)	1,095,424
	Cases per 100,000	1033 (852–1209)	688 (531–881)	554 (413–704)	1745 (1378–2119)	780 (655–915)	424 (273–616)	5,224
Europe and Central Asia	Number	734399 (613799–844935)	442598 (344672–561172)	325486 (254438–405246)	968321 (759685–1177813)	288885 (241844–341950)	129066 (85207–183064)	2,888,755
	Cases per 100,000	1387 (1159–1596)	836 (651–1060)	615 (481–766)	1829 (1435–2224)	546 (457–646)	244 (161–346)	5,457
East Asia and the Pacific	Number	3438113 (2956219–3913264)	1297733 (1038429–1626274)	823451 (609592–1057118)	2727757 (2193412–3288485)	655238 (536229–777688)	494302 (328841–691713)	9,436,594
	Cases per 100,000	2321 (1996–2642)	876 (701–1098)	556 (412–714)	1842 (1481–2220)	443 (362–525)	334 (222–467)	6,372
Latin America and the Caribbean	Number	955072 (815257–1083241)	537396 (425765–677756)	407933 (314902–517147)	920348 (751406–1093649)	235268 (195519–280124)	164888 (109929–235240)	3,220,905
	Cases per 100,000	1810 (1545–2052)	1018 (807–1284)	773 (597–980)	1744 (1424–2072)	446 (371–531)	313 (209–446)	6,104
South Asia	Number	3874622 (3255133–4475660)	1957304 (1557653–2441206)	1125281 (800287–1471321)	10126841 (7607751–12675553)	618664 (507916–741150)	203205 (131076–295087)	17,905,917
	Cases per 100,000	2245 (1886–2593)	1134 (903–1415)	652 (464–853)	5866 (4407–7343)	359 (295–430)	118 (76–171)	10,374
Middle East and North Africa	Number	488204 (402462–571691)	484094 (386408–607876)	349893 (273786–431567)	1495407 (1130592–1865688)	163087 (134699–194489)	93415 (62260–134040)	3,074,100
	Cases per 100,000	1117 (921–1308)	1107 (884–1390)	801 (627–987)	3420 (2586–4266)	373 (308–445)	214 (143–307)	7,032
Sub-Saharan Africa	Number	4430374 (3751757–5105139)	1060410 (858727–1318551)	1281867 (971635–1601732)	4380762 (3367541–5401625)	785503 (649093–939682)	192373 (125070–284836)	12,131,289
	Cases per 100,000	2591 (2194–2985)	620 (503–771)	750 (569–937)	2562 (1969–3159)	460 (380–550)	113 (74–167)	7,096
Western Sub-Saharan Africa	Number	2056777 (1733992–2379728)	486542 (396448–600924)	535773 (412020–676979)	1569783 (1214410–1929542)	334776 (276395–400912)	83100 (53414–123565)	5,066,751
	Cases per 100,000	2828 (2385–3272)	669 (546–827)	737 (567–931)	2159 (1670–2653)	461 (381–552)	115 (74–170)	6,969
Central Sub-Saharan Africa	Number	476231 (403059–544899)	105598 (83203–135057)	159777 (112730–212478)	569843 (436073–705495)	94749 (77403–113592)	22322 (14378–32171)	1,428,520
	Cases per 100,000	2301 (1948–2633)	511 (402–653)	772 (545–1027)	2753 (2107–3409)	458 (374–549)	108 (70–156)	6,903
Eastern Sub-Saharan Africa	Number	1622747 (1369967–1865589)	358640 (289879–446935)	489671 (362217–625240)	1824540 (1391859–2248468)	299975 (247740–358721)	68924 (44638–101370)	4,664,497
	Cases per 100,000	2531 (2136–2909)	560 (452–697)	764 (565–975)	2845 (2171–3506)	468 (387–560)	108 (70–159)	7,276
Southern Sub-Saharan Africa	Number	214764 (182842–247327)	55604 (44030–69664)	63914 (44593–86373)	154615 (122342–186165)	37204 (30649–44371)	8776 (5687–13128)	534,877
	Cases per 100,000	2653 (2259–3055)	687 (544–861)	790 (551–1067)	1910 (1512–2300)	460 (379–549)	109 (71–163)	6,609
Global	Number	14148322 (12036835–16216298)	5928288 (4749336–7364009)	4433545 (3376788–5567220)	20998409 (16142819–25947466)	2912437 (2418074–3461585)	1367582 (898677–1947054)	49,788,583
	Cases per 100,000	2135 (1816–2447)	895 (717–1111)	669 (510–840)	3168 (2436–3915)	440 (365–523)	207 (136–294)	7,514

* 95% uncertainty intervals not available for all disabilities.

**Table 4:** Global and regional prevalence estimates (95% uncertainty intervals) of disabilities among children aged 5 to 19 years from GBD 2019.

**Region**	**Metric**	**Hearing loss**	**Vision loss**	**Epilepsy**	**Developmental intellectual disability**	**Autism spectrum disorders**	**Attention-deficit/hyperactivity disorders**	Total^*^
North America	Number	1226942 (1069237–1389908)	829331 (673427–1015300)	464116 (377040–566934)	1127243 (881538–1386467)	509875 (428720–599971)	3365797 (2261084–4803878)	7,523,304
	Cases per 100,000	1780 (1551–2016)	1203 (977–1473)	674 (547–823)	1635 (1279–2011)	740 (622–871)	4882 (3280–6967)	10,914
Europe and Central Asia	Number	4583297 (4026175–5148943)	2222942 (1818252–2714517)	1158295 (896858–1486995)	2744266 (2120532–3364352)	838512 (701345–993733)	4550322 (3110708–6365862)	16,097,634
	Cases per 100,000	2843 (2498–3194)	1379 (1128–1684)	719 (557–923)	1703 (1316–2087)	521 (436–617)	2823 (1930–3949)	9,988
East Asia and the Pacific	Number	22128237 (19553458–24940194)	5991479 (4904617–7257251)	2552101 (1987366–3300801)	7356504 (5854718–8964365)	1770957 (1461017–2120794)	15649369 (10849799–21465647)	55,448,647
	Cases per 100,000	5181 (4578–5840)	1403 (1149–1700)	598 (466–773)	1723 (1371–2099)	415 (343–497)	3664 (2541–5026)	12,984
Latin America and the Caribbean	Number	6648994 (5902702–7477938)	2930755 (2386395–3569863)	1438411 (1127718–1831419)	2550241 (2058769–3060952)	670370 (555365–798010)	6064849 (4188888–8551760)	20,303,620
	Cases per 100,000	4153 (3687–4671)	1831 (1491–2230)	899 (705–1144)	1593 (1286–1912)	419 (347–499)	3788 (2617–5342)	12,683
South Asia	Number	28211185 (24273897–32232474)	8138965 (6764205–9851146)	4151288 (3099984–5359019)	30468226 (22914705–38180927)	1834585 (1512061–2209420)	7824749 (5139601–11183238)	80,628,998
	Cases per 100,000	5126 (4410–5856)	1479 (1229–1790)	755 (564–974)	5536 (4163–6937)	334 (275–402)	1422 (934–2032)	14,652
Middle East and North Africa	Number	2813081 (2427661–3208346)	2477027 (2036894–2992981)	957295 (764983–1203938)	4016620 (3022254–5024694)	435499 (359818–520101)	3169552 (2163344–4468032)	13,869,074
	Cases per 100,000	2258 (1949–2575)	1988 (1635–2403)	769 (614–967)	3224 (2426–4033)	350 (289–418)	2544 (1737–3586)	11,133
Sub-Saharan Africa	Number	22442961 (19306417–25523614)	4073575 (3435394–4846169)	3459890 (2673539–4485332)	9863944 (7536483–12220930)	1807381 (1487757–2159942)	5801510 (3840848–8271417)	47,449,261
	Cases per 100,000	5314 (4571–6043)	965 (814–1148)	820 (633–1062)	2336 (1785–2894)	428 (353–512)	1374 (910–1959)	11,237
Western Sub-Saharan Africa	Number	10039827 (8585704–11437723)	1772251 (1501258–2105376)	1358388 (1023033–1787672)	3372979 (2584303–4197495)	750778 (618519–898133)	2446774 (1607405–3493043)	19,740,997
	Cases per 100,000	5720 (4891–6516)	1010 (856–1200)	774 (583–1019)	1922 (1473–2392)	428 (353–512)	1394 (916–1990)	11,248
Central Sub-Saharan Africa	Number	2440218 (2112922–2752972)	412296 (342047–495657)	448016 (286390–650824)	1279151 (971797–1596102)	215881 (176255–257883)	655630 (431619–945625)	5,451,192
	Cases per 100,000	4836 (4188–5456)	818 (678–983)	888 (568–1290)	2535 (1926–3164)	428 (350–512)	1300 (856–1874)	10,805
Eastern Sub-Saharan Africa	Number	8396926 (7232002–9572424)	1380137 (1157587–1644255)	1370261 (1044622–1785873)	4146836 (3166775–5147128)	696321 (574120–831487)	2096146 (1388361–3004361)	18,086,627
	Cases per 100,000	5269 (4538–6006)	866 (727–1032)	860 (656–1121)	2602 (1987–3230)	437 (361–522)	1316 (872–1885)	11,350
Southern Sub-Saharan Africa	Number	1233551 (1062885–1402959)	241357 (200575–289619)	202537 (153864–262472)	400906 (317513–486844)	96865 (79345–115927)	297468 (195820–427506)	2,472,684
	Cases per 100,000	5479 (4721–6231)	1072 (891–1287)	900 (684–1166)	1781 (1411–2163)	431 (353–515)	1322 (870–1899)	10,985
Global	Number	88121532 (76891578–99618793)	26684718 (21991143–32187072)	14192633 (11172414–18071433)	58160929 (44335927–72217829)	7873281 (6532083–9413240)	46477791 (31750591–64830750)	241,510,884
	Cases per 100,000	4599 (4013–5199)	1393 (1148–1680)	741 (583–943)	3035 (2314–3769)	411 (341–492)	2426 (1657–3383)	12,605

* 95% uncertainty intervals not available for all disabilities.

**Table 5:** Global and regional prevalence estimates (95% uncertainty intervals) of disabilities among children younger than 20 years from GBD 2019.

**Region**	**Metric**	**Hearing loss**	**Vision loss**	**Epilepsy**	**Developmental intellectual disability**	**Autism spectrum disorders**	**Attention-deficit/hyperactivity disorders**	Total^*^
North America	Number	1443622 (1260095–1623672)	973597 (801351–1171707)	580289 (474814–694717)	1493177 (1172810–1827421)	673405 (566292–791760)	3454636 (2317514–4930022)	8,618,726
	Cases per 100,000	1606 (1402–1806)	1083 (892–1303)	646 (528–773)	1661 (1305–2032)	749 (630–881)	3842 (2577–5482)	9,587
Europe and Central Asia	Number	5317696 (4661136–5957133)	2665540 (2220754–3201168)	1483781 (1161312–1851246)	3712587 (2885054–4541304)	1127397 (943188–1336345)	4679388 (3206381–6539742)	18,986,389
	Cases per 100,000	2483 (2177–2782)	1245 (1037–1495)	693 (543–865)	1734 (1347–2121)	527 (441–624)	2185 (1497–3054)	8,867
East Asia and the Pacific	Number	25566349 (22666249–28615270)	7289211 (6079418–8726594)	3375552 (2681974–4248571)	10084260 (8052049–12264190)	2426195 (1995116–2897387)	16143670 (11192831–22148095)	64,885,237
	Cases per 100,000	4445 (3941–4975)	1268 (1057–1517)	587 (467–739)	1753 (1400–2132)	422 (347–504)	2807 (1946–3850)	11,282
Latin America and the Caribbean	Number	7604066 (6768579–8453553)	3468151 (2896484–4159632)	1846344 (1478702–2287072)	3470589 (2818649–4153597)	905637 (753110–1077016)	6229737 (4299528–8776093)	23,524,524
	Cases per 100,000	3572 (3180–3971)	1629 (1361–1954)	868 (695–1075)	1631 (1324–1951)	426 (354–506)	2927 (2020–4123)	11,053
South Asia	Number	32085806 (27728413–36385664)	10096269 (8439115–11984658)	5276568 (4094348–6599348)	40595067 (30539944–50842037)	2453248 (2019630–2962215)	8027954 (5266209–11471660)	98,534,912
	Cases per 100,000	4438 (3835–5032)	1397 (1168–1658)	730 (567–913)	5615 (4224–7032)	340 (280–410)	1111 (729–1587)	13,631
Middle East and North Africa	Number	3301284 (2862269–3746353)	2961120 (2494463–3525529)	1307187 (1063318–1619453)	5512026 (4148436–6892949)	598586 (495243–713797)	3262966 (2224812–4598774)	16,943,169
	Cases per 100,000	1962 (1701–2226)	1760 (1482–2095)	777 (632–963)	3275 (2465–4095)	356 (295–425)	1939 (1322–2732)	10,069
Sub-Saharan Africa	Number	26873334 (23225530–30370158)	5133984 (4380184–6041603)	4741756 (3782633–5953097)	14244706 (10926006–17570979)	2592883 (2136090–3099711)	5993883 (3966944–8542723)	59,580,546
	Cases per 100,000	4529 (3914–5118)	866 (739–1019)	800 (638–1004)	2401 (1842–2961)	437 (360–523)	1010 (669–1440)	10,043
Western Sub-Saharan Africa	Number	12096603 (10409381–13721117)	2258793 (1943559–2638917)	1894161 (1469697–2416851)	4942762 (3802172–6119971)	1085553 (896738–1299438)	2529873 (1663706–3611220)	24,807,745
	Cases per 100,000	4873 (4193–5527)	910 (783–1063)	763 (592–974)	1991 (1532–2466)	438 (362–524)	1019 (671–1455)	9,994
Central Sub-Saharan Africa	Number	2916448 (2512575–3258254)	517894 (433288–616952)	607792 (401690–844674)	1848993 (1407885–2300062)	310629 (253551–371262)	677951 (445679–977406)	6,879,707
	Cases per 100,000	4099 (3531–4579)	728 (609–867)	855 (565–1187)	2599 (1979–3233)	437 (357–522)	953 (627–1374)	9,671
Eastern Sub-Saharan Africa	Number	10019673 (8666559–11379839)	1738777 (1488881–2044281)	1859931 (1438907–2348494)	5971376 (4562094–7412340)	996295 (821527–1190208)	2165070 (1432587–3105380)	22,751,122
	Cases per 100,000	4483 (3878–5092)	778 (667–915)	833 (644–1051)	2672 (2041–3317)	446 (368–533)	969 (641–1390)	10,181
Southern Sub-Saharan Africa	Number	1448315 (1250897–1636349)	296961 (249538–352908)	266451 (207123–340369)	555520 (438187–673890)	134069 (109930–160120)	306243 (201417–440151)	3,007,559
	Cases per 100,000	4731 (4087–5346)	971 (816–1153)	871 (677–1112)	1815 (1432–2202)	438 (360–524)	1001 (658–1438)	9,827
Global	Number	102269853 (89657165–115064557)	32613006 (27412553–38676284)	18626177 (15136201–23044362)	79159337 (60490508–98168458)	10785718 (8953061–12859912)	47845372 (32634830–66892474)	291,299,463
	Cases per 100,000	3966 (3477–4462)	1265 (1063–1500)	723 (587–894)	3070 (2346–3807)	419 (348–499)	1855 (1266–2594)	11,298

* 95% uncertainty intervals not available for all disabilities.

**Figure 1:**
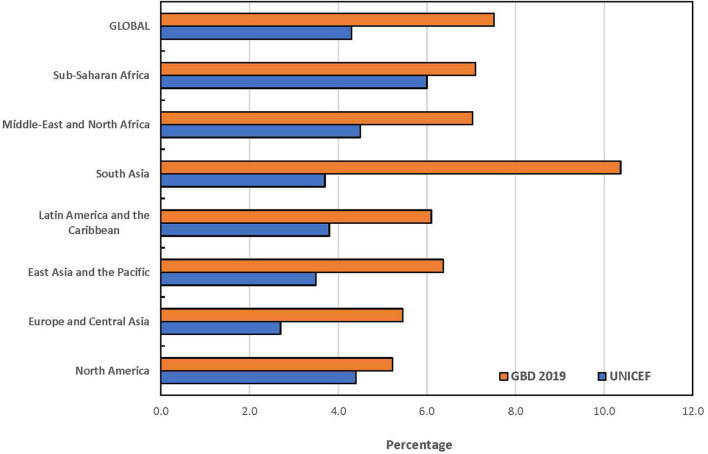
Prevalence estimates of disabilities among children under 5 years by UNICEF and GBD 2019.

**Figure 2:**
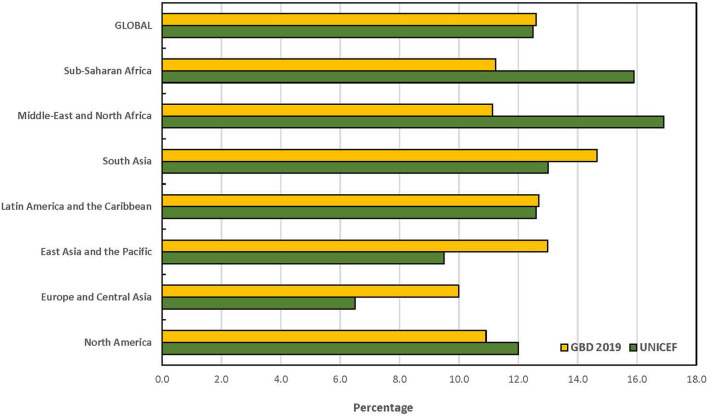
Prevalence estimates of disabilities among children aged 5 to 19 years by UNICEF and GBD 2019.

**Figure 3:**
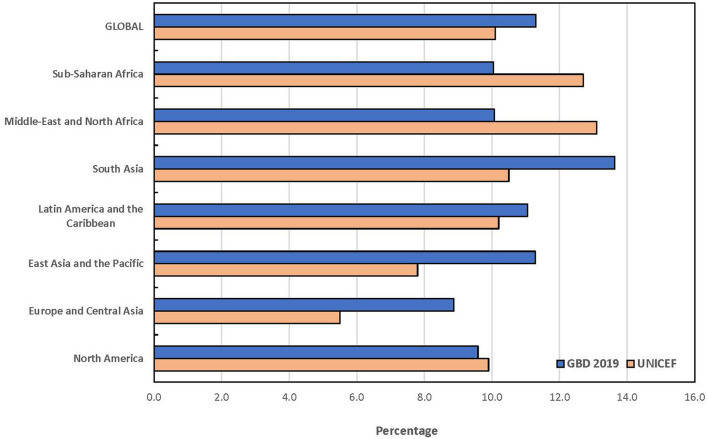
Prevalence estimates of disabilities among children under 20 years by UNICEF and GBD 2019.

**Figure 4:**
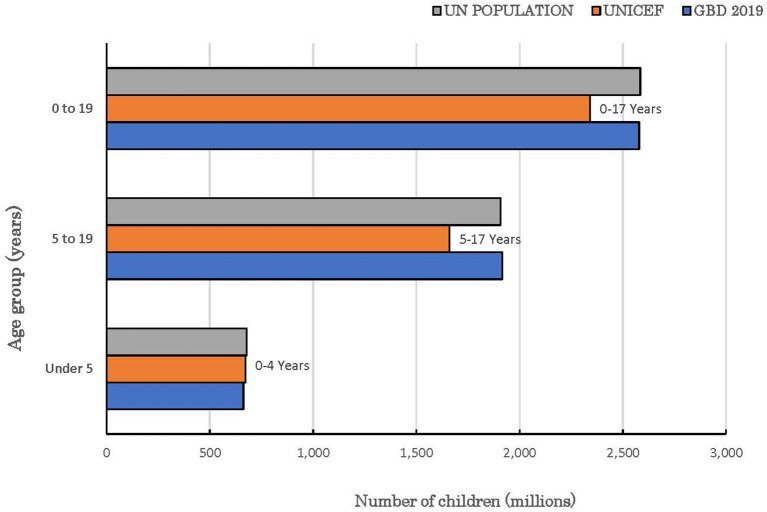
Global population of children with and without disabilities in the reported age groups.

The statistical comparison of the estimates from both UNICEF and GBD is summarized in Table 6. The *t*-tests for the overall mean for each age group only showed statistically significant difference among children under 5 years (*p* = 0.003). At a ±3 percentage point margin, the TOST showed that the estimates from both sources were statistically equivalent, except for children under 5 years (*p* = 0.375). None of the global and regional prevalence ratios for children under 5 years fell within the goodness-of-fit criteria while all absolute differences for the combined category of children under 20 years fell within goodness-of-fit criteria. The goodness-of-fit criteria were met in North America, Latin America and the Caribbean, Sub-Saharan Africa and globally for all age categories except for children under 5 years. The largest absolute difference in estimates globally was recorded among children under 5 years. The regional pattern of the global estimates of children under 5 years with disabilities is also presented in Figure 1. The largest contributor to the difference between both data sources was South Asia where a 6.7 percentage point difference was recorded, and the GBD estimate was almost 3-fold of the estimate by UNICEF. Similar data for the other age groups are presented in Figures 2, 3. The populations of children in each group used by UNICEF and GBD for estimating the total number of children with disabilities globally are summarized in Figure 4.

**Table 6:** Comparison of global and regional prevalence estimates (%) between UNICEF 2022 and GBD 2019.

	**UNICEF**			**GBD 2019**			**Prevalence ratio: Criterion 0.85 to 1.15**			**Absolute difference: Criterion** ±**5**		
**Region**	** < 5 years**	**5 to 17 years**	** < 18 years**	** < 5 years**	**5 to 19 years**	** < 20 years**	** < 5 years**	**5 to 19 years**	** < 20 years**	** < 5 years**	**5 to 19 years**	** < 20 years**
North America	4.4	12.0	9.9	5.2	10.9	9.6	1.2	**0.9**	**1.0**	**0.8**	–**1.1**	–**0.3**
Europe and Central Asia	2.7	6.5	5.5	5.5	10.0	8.9	2.0	1.5	1.6	**2.8**	**3.5**	**3.4**
East Asia and the Pacific	3.5	9.5	7.8	6.4	13.0	11.3	1.8	1.4	1.4	**2.9**	**3.5**	**3.5**
Latin America and the Caribbean	3.8	12.6	10.2	6.1	12.7	11.1	1.6	**1.0**	**1.1**	**2.3**	**0.1**	**0.9**
South Asia	3.7	13.0	10.5	10.4	14.7	13.6	2.8	**1.1**	1.3	6.7	**1.7**	**3.1**
Middle East and North Africa	4.5	16.9	13.1	7.0	11.1	10.1	1.6	**0.7**	**0.8**	**2.5**	−5.8	–**3.0**
Sub-Saharan Africa	6.0	15.9	12.7	7.1	11.2	10.0	1.2	**0.7**	**0.8**	**1.1**	–**4.7**	–**2.7**
Global	4.3	12.5	10.1	7.5	12.6	11.3	1.7	**1.0**	**1.1**	**3.2**	**0.1**	**1.2**

t-Test for statistical difference in overall mean in each age group: < 5 years (t = 4.185, df = 11.4, p = 0.001); 5 to 19 years (t = 0.264, df = 9.71, p = 0.797); < 20 years (t = 0.746, df = 11.37, p = 0.471).

Two one-sided test (TOST) for statistical equivalence of overall mean in each age group, GBD vs UNICEF: < 5 years (6.9% vs 4.1%, p = 0.375); 5 to 19 years (12.0 vs. 12.4%, p = 0.0.032); < 20 years (10.7 vs. 10.0%, p = 0.024).

## Discussion

It is important to clarify the significance of the findings on the prevalence estimates reported from different databases against the backdrop of the adverse consequences confronting children with disabilities over the life course (10–12, 17, 29, 30). Globally, the likelihood of a surviving child having a disability is estimated to be at least 10 times higher than that of dying before their fifth birthday (29). When compared to children without disabilities, children with disabilities are 42% less likely to have foundational reading and numeracy skills, 49% more likely to have never attended school, 47% more likely to drop out of primary school and 20% less likely to have expectations of a better life (17). Available reports also suggest that between 80 and 90% of people with disabilities of working age are likely to be unemployed in low- and middle-income countries compared to between 50 and 70% in high-income countries (30). Given the peculiar challenges often associated with measuring disability across various functional domains (4, 8), our primary goal was to examine the degree of alignment between the reported estimates from data sources that rely on different methodologies with a view to highlighting areas for further consideration.

A key finding in this study is that available prevalence estimates of children with disabilities from UNICEF and GBD appear complementary and emphasize the need for appropriate policy interventions from early childhood. The comparability of the prevalence estimates of disabilities among all children and adolescents as a group, despite the differences in the approaches to estimation is noteworthy. The GBD estimate of all children with mild-to-moderate disabilities exceeded the estimate of moderate-to-severe disabilities from UNICEF by 55 million or 23.2%. This variance can be attributed to several factors. Firstly, the UNICEF estimates excluded children aged 18 and 19 years. The inclusion of these children by GBD is consistent with the adolescent age group used by the UN Population Division (25) and the United Nations Inter-Agency Group for Child Mortality Estimation that comprises the UN, UNICEF, WHO and the World Bank (2). It is unclear why this group of children was excluded in the substantive survey tool designed by the Washington Group that was adopted by UNICEF. Secondly, the population of all children in each group that served as denominator for computing the estimated prevalence differed. For example, the world population of children 0–19 years in 2019 by the UN Population Division was ~2.6 billion (25), same as the GBD denominator for estimating the prevalence for this age group. In contrast, the population of children aged 0–17 years and 5–17 years used as denominator by UNICEF was 2.3 billion and 1.7 billion, respectively. If the prevalence of 12.5% for children aged 5–17 years reported by UNICEF were applied to the 1.9 billion children aged 5–19 years by UN, the prevalence of disabilities among all children (0–19 years) would have increased to ~266 million compared to 291 million by GBD 2019. Thirdly, the reported estimates by UNICEF excluded mild disabilities in all age groups. However, mild disabilities are always significantly more prevalent than moderate-to-severe disabilities regardless of the approach to measurement (4). It is understandable that the child functioning module is likely to produce spurious findings as it relies entirely on subjective assessment by respondents. It is, therefore, not unlikely that children who have mild activity limitations might not be reported as having a disability while some children without disability may also be erroneously reported as disabled (31). However, the decision to exclude mild disabilities is inconsistent with the ICF provisions which recognize that the affected children may encounter functional difficulties under different environmental conditions (7). For example, children with minimal hearing loss (comprising slight or mild bilateral and unilateral hearing impairments), are frequently associated with adverse effects across different functional domains including speech and language development, academic performance, and social interactions (32, 33).

The significant disparities in the prevalence estimates among children under 5 years also merit clarification because of their special relevance to the subsisting global commitments for early childhood development under the SDGs (4.2.1) for this age group (1, 21, 29). The child functioning module used in country surveys excluded children under 2 years because of the challenges in eliciting functional limitations reliably through parental response. Usually, the effects of some impairments in infants may not be apparent to the parents because they are too young to have developed the ability to carry out activities that are normal for older children. However, UNICEF recognizes that data for this age group is vital and opted to assume that the estimate for children under 2 years could be informed by the estimate for children aged 2 to 4 years in each country (17). However, this imputation does not adequately reflect the evidence on the magnitude of the incidence of neurodevelopmental impairments associated with the perinatal disorders, especially in low- and middle-income countries where perinatal care is poor (34). For example, both UNICEF and GBD agree that Sub-Saharan Africa and South Asia are associated with the poorest maternal and child health complications and remain the largest contributors to disabilities among children globally. Moreover, very limited evidence exists on the validation of the child functioning module among a large sample of children 2–4 years compared to older children in these high burden regions (35, 36). The true global prevalence of children under 5 years with disabilities is therefore likely to be closer to the GBD 2019 estimate of 50 million approximately.

Considering the peculiar challenges in disability measurement, estimates of disabilities using different approaches must necessarily be evaluated within the context of the intended purpose. UNICEF data is aimed at identifying children with functional limitations over a pre-specified range of domains as part of national population censuses and surveys. The UNICEF data also uniquely provide insights into the performance of these children across key indicators of early child development compared to children without disabilities. However, the estimates are not intended to provide information on the diagnostic entities underlying the survey responses based on the available ICD codes. Attempts to use survey responses, for example, as a first stage screening to identify people with clinical impairments, service and assistive product referral needs in four functional domains (vision, hearing, mobility, and cognition) have been shown to be associated with less-than-optimal sensitivity and specificity (37). In fact, UNICEF specifically stated that the results should not be used to assess the epidemiological characteristics of any disease or impairment but an indication of the prevalence of moderate to severe functional difficulties that, in interaction with various barriers, can place children at increased risk for non-participation and exclusion (17). In contrast, the GBD primarily sets out to quantify the long-term sequelae associated with diverse health conditions based on ICD codes to inform appropriate interventions (primary, secondary and tertiary prevention) within the healthcare systems. The estimates provide information on the scope, nature and magnitude of the rehabilitation services that are required to support children with specific disabilities. While the GBD estimates do not cover all known disabilities, they are notably consistent with the recognition of specific diagnostic disability entities under the US' Individuals with Disabilities Education Act (IDEA) 2004 (38) and the UK Equality Act 2010 (39). Additionally, the ICF views disability as an umbrella term for impairments, activity limitations, and participation restrictions and denotes the negative aspects of the interaction between an individual (with a health condition) and that individual's contextual factors (environmental and personal factors) (7). The ICF also underscores its complementarity with the ICD diagnostic entities.

Disability measurement is frequently linked with models for conceptualizing disability (17, 40–43). The predominant and oldest model - the medical or biomedical model - defines disability primarily as a medical condition resulting from some physiological impairment that can either be prevented or managed to optimize individual functioning (17, 40, 41). The social model emerged in the 1970's to present disability as not due to an individual pathology but as a failure of the policy, cultural and physical environments to accommodate differences in function (42). Unfortunately, the social model evolved from a narrow and restricted conceptualization of disability beyond physical impairment (41, 42). The biopsychosocial model was later introduced to address the limitations of the medical model in recognizing the psychological, social, and behavioral dimensions of a medical condition (43, 44), and became the focus of the ICF. However, the ICF was never intended to replace the medical model but to enhance it (45). While it may be easier to elicit functional difficulties through household surveys, such responses do not provide a pathway for the effective care of children with disabilities within the health systems (37). In fact, it is difficult to identify children with self-limiting constitutional developmental delays based on survey responses. Any suggestion that these models of disability are mutually exclusive is therefore erroneous, counter-productive, and inconsistent with the ICF principles embraced by UNICEF (40–42). For example, routine screening and confirmation of babies for congenital hearing impairment is legally mandatory within the first 3 months of life in many high-income countries well-before the functional difficulties associated with hearing impairment become apparent usually after 18–24 months (46). Functional approach to prevalence estimate will miss such infants. No single approach to prevalence estimation is flawless, better, or sufficient by itself to serve the multidimensional interests of children with disabilities. This fact is duly acknowledged by UNICEF and GBD (13, 17, 21, 22). The ongoing implementation of the MDS tool designed to elicit information on functional limitations and associated health conditions by WHO and the World Bank (18) is likely to offer a more robust comparative analysis of prevalence estimates in future.

Additionally, neither UNICEF nor GBD cover the full spectrum of known disabilities in children. Thus, the reported prevalence should appropriately be regarded as the minimum estimates among children with disabilities. All estimation approaches require some degree of imputation and statistical adjustments, and concerns have been raised on modeling approaches in general and particularly for those used by GBD (6, 47). While efforts to improve the reliability of such estimates are needed, the COVID-19 pandemic has further underscored the need for different approaches to prevalence estimation outside the traditional in-person house-to-house surveys.

The focus of this paper was to examine how the available global and regional estimates of disabilities among children can be optimized to facilitate the implementation of policies and action plans for achieving inclusive education as envisioned in the SDGs and reinforced by CRPD (2, 9). In our view, the estimates from both sources, using functional approach and the identification of specific impairments associated with various health conditions should be regarded as complementary and in line with the ICF framework. While an effort by UNICEF to include children younger than 2 years through data imputation based on findings among children 2–4 years is commendable, we wish to reiterate earlier calls on the need to expand the CFM to include children younger than 2 years in line with the principles and concept of early childhood development globally (48). This is not only consistent with the spirit and letter of the SDG of leaving no child behind, but also allows for improved age-specific comparison across all databases. Additionally, there is need to highlight the inequalities among children and adolescents with disabilities in low- and middle-income countries compared to high-income countries across all data sources and indicators of functioning status which are required for any effective rights-based advocacy.

Some limitations of this study are worthy of emphasis. For example, the age range covered by both data sources differed and the lack of adequate validation studies for child functioning module for children under 5 years would have compromised the estimates by UNICEF as reference standards for assessing data from other sources. Our inability to obtain 95% uncertainty intervals for the combined estimates of the six disabilities included in the GBD 2019 as at the time of this study is a limitation that can be resolved in future with additional inputs from the organization. Notwithstanding, the overarching evidence from the available data sources demonstrate the magnitude of disabilities among children and adolescents that need to be addressed within the SDGs framework to ensure improved developmental trajectory for the affected children from early childhood for optimal educational opportunities.

## Conclusion

The global and regional prevalence estimates of children and adolescents younger than 20 years with disabilities relevant to the monitoring requirements of the SDGs are now provided by UNICEF and GBD. The latest prevalence estimates of disabilities reported from these two sources are generally comparable but would require improved alignment of the age groups and the selected severity thresholds, especially for children under 5 years. The ICF conceptually encapsulates the medical and social models of disability, and no single data source presently fully satisfies the biophysiological paradigm of this framework. While the UNICEF data provides unique and valuable insights on the functional challenges faced by children with disabilities compared to children without disabilities, the GBD data offer equally valuable insights on the nature of the medical services that will assist these children optimize their functional performance. We conclude that the interests of children with disabilities and their families will continue to be well-served by data from a variety of complementary sources to inform global policy interventions. Future analysis is likely to be boosted by the inclusion of findings from the ongoing MDS implementation by WHO and the World Bank.

## Data availability statement

The original contributions presented in the study are included in the article/Supplementary material, further inquiries can be directed to the corresponding author/s.

## Author contributions

BO drafted the manuscript. VK, AS, FO, and AD critically reviewed the draft, contributed to the statistical analysis, and suggested essential edits. BO and AD are guarantors. All authors contributed to revising the manuscript, and approved the final version as submitted.

## Conflict of interest

The authors declare that the research was conducted in the absence of any commercial or financial relationships that could be construed as a potential conflict of interest.

## Publisher's note

All claims expressed in this article are solely those of the authors and do not necessarily represent those of their affiliated organizations, or those of the publisher, the editors and the reviewers. Any product that may be evaluated in this article, or claim that may be made by its manufacturer, is not guaranteed or endorsed by the publisher.
