# Supplementary figures and images for: Global and regional prevalence of disabilities among children and adolescents: Analysis of findings from global health databases
Source: Front Public Health. 2022 Sep 23;10:977453. doi: 10.3389/fpubh.2022.977453 (PMC9554924; doi:10.3389/fpubh.2022.977453)

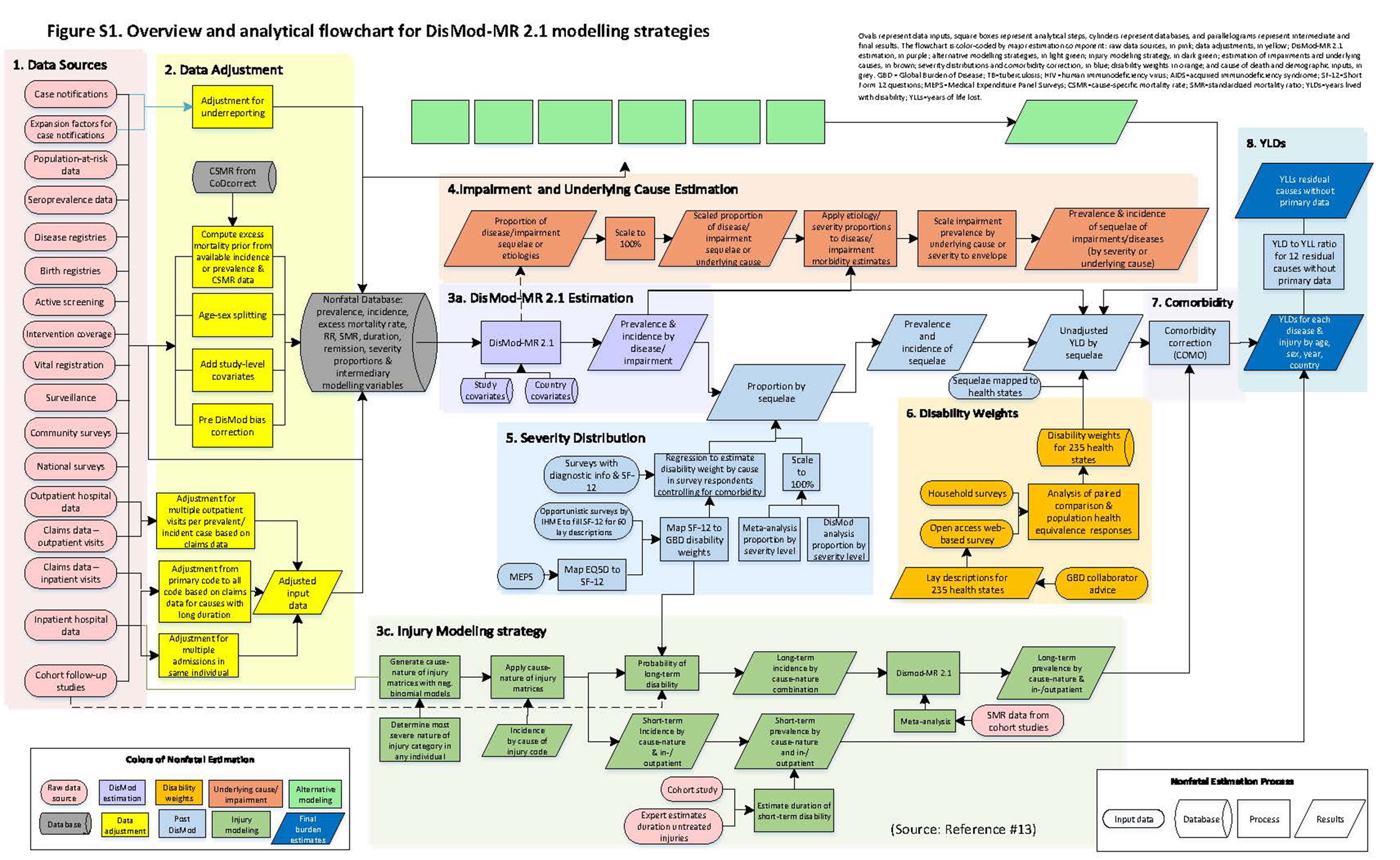

Supplement: Supplementary file 1 [file Image_1.jpg]
